# Can Brain-Derived Neurotrophic Factor Be Considered a Biomarker for Bipolar Disorder? An Analysis of the Current Evidence

**DOI:** 10.3390/brainsci13081221

**Published:** 2023-08-20

**Authors:** Gianmarco De Felice, Mario Luciano, Alessia Boiano, Giulia Colangelo, Pierluigi Catapano, Bianca Della Rocca, Maria Vita Lapadula, Elena Piegari, Claudia Toni, Andrea Fiorillo

**Affiliations:** Department of Psychiatry, University of Campania Luigi Vanvitelli, 80138 Naples, Italy; gianm.defe@gmail.com (G.D.F.); alessiaboiano20@gmail.com (A.B.); giulia.colangelo@gmail.com (G.C.); dr.pierluigi.catapano@gmail.com (P.C.); dellarocca.bianca@gmail.com (B.D.R.); lapadulamariavita@yahoo.it (M.V.L.); elena.piegari@unicampania.it (E.P.); claudiatoni96@gmail.com (C.T.); andrea.fiorillo@unicampania.it (A.F.)

**Keywords:** brain-derived neurotrophic factor, BDNF, bipolar disorder

## Abstract

Brain-derived neurotrophic factor (BDNF) plays a key role in brain development, contributing to neuronal survival and neuroplasticity. Previous works have found that BDNF is involved in several neurological or psychiatric diseases. In this review, we aimed to collect all available data on BDNF and bipolar disorder (BD) and assess if BDNF could be considered a biomarker for BD. We searched the most relevant medical databases and included studies reporting original data on BDNF circulating levels or Val66Met polymorphism. Only articles including a direct comparison with healthy controls (HC) and patients diagnosed with BD according to international classification systems were included. Of the 2430 identified articles, 29 were included in the present review. Results of the present review show a reduction in BDNF circulating levels during acute phases of BD compared to HC, which increase after effective therapy of the disorders. The Val66Met polymorphism was related to features usually associated with worse outcomes. High heterogeneity has been observed regarding sample size, clinical differences of included patients, and data analysis approaches, reducing comparisons among studies. Although more studies are needed, BDNF seems to be a promising biomarker for BD.

## 1. Introduction

Bipolar disorder (BD) is a severe mental disorder with a reported prevalence in the general population of 2.4% [1,2]. People suffering from BD report a worsening of social functioning in terms of work-related problems, a high college drop-out rate, and interpersonal difficulties [3,4,5], as well as increased comorbidity with physical disorders and mortality compared to the general population [6,7,8,9]. In 2019, BD resulted in 8.50 million global disability-adjusted life years (DALYs), equivalent to 0.3% of DALY, contributing to 6.8% of DALYs for aggregate mental disorders [1]. Moreover, BD is associated with a significant economic burden [10], with an estimated total annual national economic expenditure of more than USD 195 billion in the US alone, with approximately 25% attributed to direct medical costs [10] and the remaining to loss of productivity.

Bipolar disorder is a multifactorial disease, with a complex interaction between biological and environmental factors, both contributing to the definition of the pathophysiology of BD [11,12,13]. Nevertheless, studies are still far from identifying biological mechanisms underlying the etiopathogenesis of BD and other mental disorders [14,15].

In the past few decades, research has been focused on the correlation between BD and neuro- and systemic inflammation [16]; in particular, the role of pro-inflammatory cytokines, immuno-modulators, and growth factors in influencing the episodic course of the disorder has been investigated [17].

Neuroinflammation is characterized by an increased number of circulating proinflammatory cytokines, increased immune cell entry into the central nervous system through the blood–brain barrier, microglial activation, and degeneration of the encephalic tissue [18]. Different brain insults can cause microglial activation, with the release of interleukin-1β (IL-1β) and activation of astrocytes, starting the pro-inflammatory cascade [19].

Many studies have shown higher levels of inflammatory markers both in the bloodstream and in cerebro-spinal fluid (CSF) in BD patients [20]. Consistent findings have been reported through the analysis of brain tissue samples of deceased BD patients [21] and with neuroimaging studies. In a positron emission tomography (PET) study, Haarman et al. (2014) [22] found a significantly increased binding potential, an indirect marker of neuroinflammation, in BD patients when compared with healthy controls (HC). Chronic neuroinflammation can lead to several modifications in brain tissue, particularly the reduction in circulating brain-derived neurotrophic factor (BDNF) [23]. BDNF is a growth factor synthesized mostly in the brain in response to neuronal activity; it is produced as pro-BDNF, a precursor that can be activated in mature BDNF (m-BDNF) by extracellular metalloproteinases or intracellular endoproteases.

BDNF explicates its function mostly by the activation of the TrkB receptor. During intrauterine life, BDNF signaling guides the differentiation from progenitors into mature brain cells [24], but it keeps its role in activating neurogenesis even in adult life [25]. BDNF plays a key role in the regulation of neuronal transmission and synaptic plasticity, operating on both the presynaptic side, acting on the neurotransmitters’ release, and the postsynaptic side, modulating receptors’ expression [26]. BDNF can also be found in the bloodstream, since it is also produced by other tissues and cells, such as cardiomyocytes and platelets [27]. Many studies have reported that changes in BDNF serum levels may reflect modifications in the brain’s BDNF production and/or clearance [28].

Recent data suggest a correlation between BDNF signaling deficits and some major brain diseases, including psychiatric disorders such as schizophrenia, major depressive disorder (MDD), and BD [29,30,31]. A significant decrease in circulating BDNF levels in BD patients compared to healthy controls, especially during acute episodes of the illness, has been found [32], suggesting its possible mediating role in affective disorders.

Moreover, a large body of literature has investigated the possible influence on BDNF activity of the rs6265 polymorphism, a single-nucleotide polymorphism (SNP) in the BDNF gene. The rs6265 polymorphism is a common functional nonsynonymous SNP, a coding mutation that changes the protein sequence, in which the amino acid valine (Val) is replaced with methionine (Met) in the BDNF protein, resulting in a less efficient BDNF secretion. Due to the important role played by BDNF in the nervous system, this SNP has been extensively studied in the pathogenesis of several psychiatric disorders, including mood disorders [33,34].

The role of BDNF in influencing both the clinical presentation and outcome of BD has been recently investigated, with conflicting results [35,36], which can be due to differences in study methodologies and sample heterogeneity regarding age and mood fluctuation. In fact, the levels of BDNF can vary in the different phases of the disorder and with the age of patients [37,38].

In this narrative review, we aim to review the current literature on the role of BDNF in BD and to investigate if it can be considered a reliable predictive biomarker for BD. To our knowledge, this is the first review to investigate both BDNF circulating levels and BDNF Val66Met polymorphism.

## 2. Materials and Methods

We searched the most relevant medical databases, including PubMed, Scopus, and Web of Science, until 11 April 2023, using as keywords “BDNF” or “brain derived neurotrophic factor” or “Neuroinflammation” or “Neurogenesis” or “inflammat*” matched with “bipolar” or “BD” or “depressive episode” or “mood” or “affective” and with “euthymia” or “stability” or “acute phase” or “affective episode” or “manic episode” or “mixed state” or “mixed episode” or “depressive episode”. Studies were included if they reported original data on BDNF circulating levels or Val66Met polymorphism in patients with BD and if they were written in English.

All articles published until April 2023 were considered. Studies including other sub-samples or other outcomes (i.e., MDD patients; other circulating factors, such as pro-inflammatory cytokines) were included only in case it was possible to extrapolate data regarding the association among BDNF circulating levels or Val66Met polymorphism and BD. We considered only articles including a direct comparison with healthy controls, and patients diagnosed with bipolar disorder whose diagnosis was made according to international classification systems, including clinical interviews that applied the criteria of the Diagnostic and Statistical Manual of Mental Disorders or the International Classification of Diseases. We obtained full reports of potentially relevant studies. Data on study design, sample characteristics, detected biomarkers, and main findings were independently extracted by five authors (A.B., G.C., G.D.F., M.V.L., C.T.).

**Figure 1:**
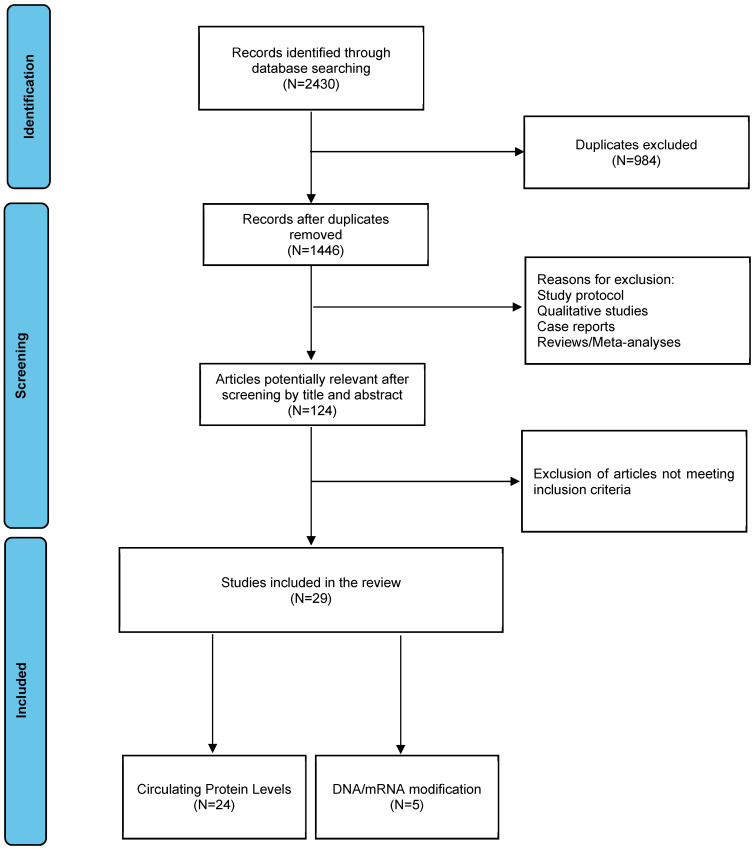
PRISMA flow diagram of selection of studies included in the review.

We identified 2430 papers, and 984 of them were excluded because of duplicates. After screening for eligibility according to inclusion criteria, 29 papers were finally included in our review (Figure 1).

Due to the methodological heterogeneity between studies, papers were subsequently grouped into two sub-categories: “BDNF/DNA modifications and circulating mRNA levels” and “Circulating BDNF protein levels”.

## 3. Results

Of the 29 papers included in the review, 5 were included in the “BDNF/DNA modification and circulating mRNA levels” group and 24 in the “Circulating BDNF protein levels” group.

About one-third of studies (9 out of 29) adopted a prospective design, and the remaining 21 studies were cross-sectional. None of the studies included in the review were randomized control trials (RCTs). Sample sizes ranged from 1446 [39] to 20 [40] participants.

### 3.1. BDNF/DNA Modifications and Circulating mRNA Levels

The five studies included in this group are reported in Table 1. All articles used real-time (RT)-PCR as a main tool. PCR is a biological technique developed in 1985 [41] to amplify a segment of DNA or RNA [42]. RT-PCR, also known as quantitative PCR, is used for simultaneous amplification and quantification of a specific DNA or RNA segment [43].

Two studies investigating BDNF mRNA levels adopted a prospective design. Li et al. (2014) [44] carried out a large prospective study involving 203 patients with a BD episode and 167 HC in whom both BDNF mRNA and BDNF serum levels were measured in a 3-year bi-annual follow-up. The authors found that BD patients had lower BDNF levels at baseline compared with healthy controls. Longer follow-ups could be useful to identify those patients experiencing a first manic or hypomanic episode in the following years.

In the second longitudinal study, Cinar RK et al. (2016) [45] assessed and compared BDNF mRNA levels in a sample of 20 BD patients during a manic episode with HC. BDNF mRNA levels were downregulated during acute manic episodes and increased in remission phases, being still lower than the levels of controls.

All other studies adopted a cross-sectional design. Soeiro-De-Souza et al. (2012) [46] and Nassan et al. (2015) [47] reported a correlation between the Val66Met polymorphism and a decrease in creativity during a manic phase; moreover, Nassan et al. (2015) [47] found that this genetic variation is more represented in early-onset BD.

Both studies have some limitations. The Soeiro-De-Souza study [46] would have had a higher impact with a larger sample and an additional measure of creativity; in the Nassan study [47], the retrospective classification of early onset BD might represent an important assessment bias.

Finally, Dell’Osso et al. (2014) [48] carried out an epigenetic study on BDNF gene promoter methylation. Methylation is a form of epigenetic gene silencing, consisting of gene inactivation [49]. They found higher BDNF gene promoter methylation levels in BD patients compared to HC and higher methylation levels during depressive phases compared with manic phases or mixed states.

### 3.2. Circulating BDNF Protein Levels

In total, 24 studies were included in this section (Table 2); most of them (17 out of 24) adopted a cross-sectional design, while the other 8 studies had a prospective design. All the studies measured circulating BDNF protein levels by ELISA, a quantitative analytical method used to measure the concentration of specific molecules. ELISA is based on the use of enzymes to show an antigen–antibody binding reaction, measured as a macroscopic color change. This technique allows us to assess very small quantities of molecules in biological fluids [50].

### 3.3. Acute Episodes of BD vs. HC

Seventeen studies have investigated the circulating levels of BDNF in BD patients during acute episodes compared with HC, and most of them have shown decreased BDNF circulating levels in BD patients during acute episodes when compared with HC. Only Barbosa et al. (2010) [51] and Poletti et al. (2017) [52] showed higher BDNF circulating levels in BD patients during acute episodes, while Binici et al. (2016) [53], Ameele et al. (2017) [54], and Skibinska et al. (2021) [55] observed no significant difference in BDNF circulating levels between acute episodes of BD patients and HC. Most studies [44,56,57,58,59,60] have investigated BDNF serum levels during a depressive episode, while Tramontina et al. (2009) [40], Machado-Vieira et al. (2007) [61], and Oliveira et al. (2009) [59] have included BD patients in a manic phase; only Piccinni et al. (2014) [60] have included 19 mixed state patients, while Tunca et al. (2014) [62] and Yoshimura et al. (2006) [63] included both manic and depressive episodes.

### 3.4. Euthymic Patients vs. HC

Monteleone et al. (2008) [64], Nuernberg et al. (2016) [65], Aas et al. (2018) [39], and Mansur et al. (2016) [66] showed a significant decrease in BDNF serum levels in BD patients compared to HC, while Dias et al. (2009) [67], Chou et al. (2012) [68], and Rosa et al. (2014) [69] did not find any significant difference between patients and healthy controls.

### 3.5. Acute Episodes vs. Euthymia

Two studies [62,70] showed a significant decrease in BDNF serum levels during acute episodes compared with euthymic phases. Similar results were reported by Zhao et al. (2017) [71], who found a lower ratio of mature BDNF to precursor BDNF in BD patients compared to HC. Moreover, Tramontina et al. (2009) [40] and Nuernberg et al. (2016) [65] have highlighted a significant increase in BDNF levels after effective treatment. On the contrary, Ameele et al. (2017) [54] and Jacoby et al. (2016) [72] found no significant difference between euthymic and acute phases of BD. When assessing the levels of BDNF in the different acute phases of BD, Yoshiumura et al. (2006) [63] observed that depressed BD patients have lower BDNF circulating levels compared to manic patients.

### 3.6. Other Findings

Cunha et al. (2006) [70] and Machado-Vieira et al. (2007) [61] found a negative correlation between BDNF levels and symptom severity. Teng et al. (2021) [57] observed a positive correlation between BDNF levels and cognitive functions in a sample of 45 BD II patients matched with 40 HC. On the other hand, Barbosa et al. (2010) [51] did not find any correlation between BDNF levels and clinical parameters, and Chou et al. (2021) [68] showed no correlation between BDNF levels and cognition in a sample of euthymic BD patients compared with HC.

Aas et al. (2018) [39] showed lower BDNF circulating levels in a large sample of acute and stable BD (n = 254) and psychotic patients (n = 254) compared to 603 HC. Interestingly, patients with a history of childhood trauma had the lowest BDNF circulating levels. On the other hand, Skibinska et al. (2021) [55] demonstrated elevated BDNF levels in patients with a family history of affective disorders.

Contrary to most findings, Barbosa et al. (2010) [51] found a higher concentration of BDNF in BD patients with a long history of disease (10 years or more), which might be due to the brain response to chronic damage or the influence on BDNF levels of the prolonged use of drugs.

As for the effect of medication on BDNF levels, Tunca et al. (2014) [62] showed a positive correlation between BDNF levels and lithium concentration in a sample of 96 BD patients, while Yoshimura et al. (2006) [63] observed no effect on BDNF levels after risperidone administration in a sample of 18 BDI patients. Oliveira et al. (2009) [59] observed significantly lower BDNF serum levels in both drug-free and treated patients during acute episodes when compared to HC. No difference between drug-free and treated patients was found.

**Table 1:** Studies on BDNF/DNA modifications and circulating mRNA levels.

	Study and Country	Study Design	Sample	Study Aim(s)	Main Findings
1	Dell’Osso, 2014, Italy [48]	Cross-sectional	144 patients (43 MDD; 61 BD I; 50 BD II); 44 HC	To investigate differences in BDNF promoter gene methylation in patients with mood disorders	Higher methylation levels in BD II (compared to BD I) and MDD patients (compared to controls). Lower methylation levels in patients during mania/mixed episodes compared to the depressive phase.
2	Li et al. 2014, China [44]	Longitudinal	203 patients with a first major depressive episode 167 HC	To explore whether BDNF levels can differentiate between MDD and bipolar disorder in the first depressive episode	At baseline, lower BDNF mRNA levels and plasma levels in BD and MDD patients compared to HC. Lower BDNF levels in the BD group compared to the MDD group. The best model for predicting BD during a first depressive episode was a combination of BDNF mRNA levels with plasma BDNF levels.
3	Nassan et al. 2015, Washington [47]	Cross-sectional	82 BD pediatric and adolescent patients 855 BD adult 764 HC	Association of BDNF Val66Met SNP with EO-BD through a case–control candidate gene study	No significant evidence of association of the minor Met allele of Val66Met with BD. Significant evidence of an association between EO-BD and Val66Met.
4	Çinar et al. 2016, Turkey [45]	Longitudinal	20 BD 20 HC	To assess the BDNF mRNA expressions in BD patients and HC	Downregulation of BDNF mRNA in mania compared to HC. In remission, increased BNDF mRNA levels, but still lower than those of the controls.
5	Soeiro-De-Souza, 2012, Brazil and USA [46]	Cross-sectional	66 BD (41 manic and 25 depressed patients) 78 HC	To determine if the Val allele is associated with increased creativity in BD	Higher BWAS scores in manic patients with the Val allele (Met−). This relationship was not observed in depressed patients or HC. No association between BDNF Met allele status and cognitive function in any of the groups.

BDNF: brain-derived neurotrophic factor; MDD: major depressive disorder; BD: bipolar disorder; EO-BD: early-onset bipolar disorder; HC: healthy controls; mRNA: messenger RNA; SNP: single-nucleotide polymorphism; Met: methionine; Val: valine; BWAS: Barron Welsh Art Scale; Val66Met: substitution of an amino acid valine with a methionine.

**Table 2:** Studies on circulating BDNF protein levels.

	Study and Country	Study Design	Sample	Study Aim(s)	Main Findings
1	Aas et al. 2018, Norway [39]	Cross-sectional	254 BD patients; 589 psychotic patients; 603 HC	To assess BDNF serum levels and to investigate if childhood trauma can affect BDNF levels	Reduced BDNF levels in patients with severe mental disorders. The most substantial reduction was observed in patients reporting childhood sexual abuse.
2	Ameele et al. 2017, Belgium [54]	Prospective case–control study	67 BD patients (35 during a depressive and 32 during a manic episode) 30 HC	To evaluate mood-specific changes in BDNF and their association with inflammatory activity	No differences in the levels of BDNF levels between BD patients and HC.
3	Barbosa et al. 2010, Brazil [51]	Cross-sectional	53 BD (34 manic and 19 euthymic) patients 38 HC	To assess BDNF levels in BD patients and their association with clinical and demographic factors	Significantly increased plasma BDNF levels in patients with mania and euthymia compared to controls, without any correlation with clinical parameters. Higher BDNF concentration in BD patients with 10 or more years of disease was found.
4	Fernandes et al. 2009, Brazil [58]	Cross-sectional	40 BD patients 10 MDD patients 30 HC	To investigate serum BDNF levels as a potential diagnostic biomarker in bipolar and unipolar depression	Lower serum BDNF levels in depressed BD patients compared to MDD patients and controls were found.
5	Binici et al. 2016, Turkey [53]	Cross-sectional	25 BD patients 17 HC	To assess BDNF serum levels in euthymic adolescents with BD type I	No difference in BDNF levels between patients and healthy controls was found.
6	Chou et al., 2021, Taiwan [68]	Cross-sectional	23 euthymic BD type I patients 33 HC	To examine the cognitive performance in euthymic BD I patients and to assess if cognitive deficits correlate with BDNF levels	No association between impaired cognition and BDNF was found.
7	Cunha et al. 2005, Brazil [70]	Cross-sectional	85 BD (32 euthymic, 21 Depressed and 32 Manic) patients 32 HC	To investigate serum BDNF levels in manic, depressed, and euthymic BD patients and in matched healthy controls	Decreased serum BDNF levels in BD patients during manic and depressive episodes compared with euthymic BD patients and HC. Negative correlation between serum BDNF levels and severity of manic and depressive symptoms was found.
8	Dias et al. 2009, Spain [67]	Cross-sectional	65 BD patients 50 HC	To investigate serum BDNF levels in BD patients compared with HC	No significant difference in serum BDNF levels in BD type I euthymic patients compared to healthy controls.
9	Jacoby et al. 2016, Denmark [72]	Longitudinal	60 BD-I patients 35 HC	To investigate whether neurotrophins and inflammatory markers vary with mood states	BDNF and the other inflammatory markers did not vary according to affective state in adjusted mixed models.
10	Machado-Vieira et al. 2007, Brazil [61]	Cross-sectional	60 BD patients 30 HC	To investigate whether BDNF levels are altered during manic phases of the disorder	Significantly decreased BDNF levels in manic patients compared to HC. Negative correlation between the severity of manic episodes and BDNF plasma levels.
11	Mansur et al., 2016, Brasile/Canada [66]	Cross-sectional	57 BD patients 26 HC	To assess the relationship between BDNF levels and indices of illness course	Lower levels of BDNF in the BD population.
12	Monteleone et al, 2008, Italy [64]	Cross-sectional	35 MDD patients 17 BD I patients 11 BD II patients 22 HC	To assess BDNF circulating levels in mood disorders; to exclude the possibility that comorbid psychiatric disorders exerted an effect on BDNF levels	Serum BDNF levels were reduced in both euthymic and depressed MDD patients as well as in euthymic patients with BD-I and BD-II. The reduction in circulating BDNF was not affected by drug treatments. Comorbid Axis I mental disorders did not influence circulating BDNF in affective patients.
13	Nuernberg et al., 2016, Brazil [65]	Longitudinal	236 patients with BD, MDD, SZ 100 HC	To evaluate BDNF levels at admission and discharge and compare them with HC To compare BDNF levels in the different SMI patients	BDNF levels persistently lower compared to HC were found.
14	Oliveira et al., 2009, Brazil [59]	Cross-sectional	22 BD unmedicated patients, 22 BD medicated patients, 22 HC	To assess whether drug-free patients have different levels of circulating serum BDNF compared to medicated BD patients and controls	Serum BDNF is decreased in drug-free and drug-treated BD subjects during manic and depressive episodes compared with HC. Similar serum BDNF levels in drug-free and medicated BD patients.
15	Piccinni et al. 2014, Italy [60]	Cross-sectional	18 depressed patients (16 BD patients, 2 MDD patients) 19 BD patients with mixed episode 15 HC	To assess BDNF plasma levels in patients with mixed episodes, and compare them with those with a depressed episode and HC	Lower BDNF levels in depressed patients compared to patients with mixed episodes, although the difference was not statistically significant.
16	Poletti et al. 2016, Italy [52]	Cross-sectional	36 BD patients 17 HC	To assess BDNF serum levels in BD patients and HC	Significantly higher serum BDNF levels in BD patients compared with HC were found.
17	Rosa et al. 2014, UK and Spain [69]	Cross-sectional	50 BD I patients 50 HC	To assess BDNF levels in BD I patients compared with HC	Similar levels of plasma BDNF levels in bipolar patients and healthy controls were found.
18	Shahyad et al. 2023, Iran [56]	Cross-sectional	30 BD patients 30 MDD patients 30 HC	To assess the discriminatory properties of BDNF levels for the differential diagnosis of BD and MDD	Lowest BDNF levels in BD compared to MDD or HC.
19	Skibinska et al. 2021, Poland [55]	Longitudinal	27 BD patients 52 MDD patients 31 HC	To assess serum levels of BDNF, proBDNF, mBDFN, and rBDNF	No significant difference in BDNF among patients and controls was found. Higher levels of BDNF in patients with a family history of affective disorders were found.
20	Teng et al, 2021, Cina [57]	Cross-Sectional	45 BD II patients 40 MDD patients 40 HC	To assess the role of BDNF in clinical and cognitive outcomes in medication-naïve patients with BD II and MDD patients	Decreased serum BDNF in MDD and BD II patients with a current depressive episode, compared to HC. BDNF and cognitive deficits are both of low efficiency in distinguishing BD II from MDD.
21	Tramontina et al, 2009, Brazil [40]	Longitudinal	10 BD I patients 10 HC	To assess changes in BDNF serum levels of BD patients during and after treatment of an acute episode	Decreased BDNF levels in BD patients during mania compared to HC. This difference was no longer significant after treatment. A sharp increase in BDNF levels after effective treatment was found. No correlation between BDNF levels either in acute episodes or after treatment with clinical scales was found.
22	Tunca et al, 2014, Turkey [62]	Cross-Sectional	96 BD patients (37 euthymic, 33 manic, 26 depressed) 61 HC	To assess BDNF levels across different episodes in bipolar disorders	Significantly lower BDNF levels during mania and depression compared to euthymic patients and HC. Positive correlation between BDNF levels and lithium levels.
23	Yoshimura et al, 2006, Japan [63]	Longitudinal	18 BD I patients (12 manic, 6 depressive) 20 HC	To investigate the efficacy of risperidone treatment for both acute manic and depressive episodes in BD	Decreased plasma levels of BDNF in depressed patients compared with manic patients or healthy controls. The administration of risperidone did not alter plasma BDNF levels.
24	Zhao et al, 2017, China [71]	Longitudinal	24 BD patients 37 MDD patients 44 HC	To investigate the role of mature BDNF (mBDNF) and its precursor (proBDNF) in distinguishing bipolar depression (BP) from MDD during acute depressive episode	Lower plasma mBDNF levels and mBDNF/proBDNF ratio in BP compared with MDD. The M/P ratio was restored to normal levels after antidepressant treatment in the MDD group.

BDNF: brain-derived neurotrophic factor; MDD: major depressive disorder; BD: bipolar disorder; HC: healthy controls; SZ: schizophrenia; SMI: severe mental illness; proBDNF: BDNF precursor; mBDNF: mature BDNF; rBDNF: mBDNF/proBDNF ratio; M/P ratio: mBDNF/proBDNF ratio.

## 4. Discussion

In the past decade, the role of BDNF in the course of BD has been investigated in several studies to gain a better understanding of the neurobiology of the disorder. Despite the large amount of available data, the pathophysiology of BD remains largely unknown [73]. Research on biological and social risk factors for severe mental disorders, particularly for BD, is far from being exhaustive [74,75]. In fact, available studies are highly heterogeneous, in terms of sample characteristics, mood states, and biological techniques.

The majority of studies included adult patients (18–65 years), while others also included adolescents [53]. Moreover, most studies recruited chronic and medicated patients, and only a few included first-onset drug-naive patients [44,45,57,59,61]. Finally, regarding sample selection, some studies included patients during acute or euthymic phases, and others enrolled BD patients regardless of mood state.

Overall, the results of our work show a reduction in BDNF circulating levels during acute phases of BD, which increase after effective therapy. When directly compared, the levels of BDNF were significantly lower in acute phases of BD compared to euthymic patients. This finding is particularly relevant considering that BDNF induces neuronal growth and survival and synaptic long-term potentiation (LTP) [26,76], inhibits the apoptosis cascade (by activating the phospholipase C-gamma), induces its own mRNA transcription (by enhancing phosphatidylinositol 3-Phosphate, PI3K), and modulates gene regulation.

The finding that BDNF levels are reduced in BD vs. healthy controls, and that they are reduced during acute phases vs. euthymia, can potentially link the BDNF levels to the pathophysiology of BD. In fact, the relationship between BDNF, BD, and neuroinflammation is well established. Guan et al. (2006) [77] induced an inflammatory response by administering lipopolysaccharide (LPS) to mice. Interestingly, the authors found a reduced BDNF mRNA in the hippocampus 4 h after the administration, and decreased BDNF circulating levels 7 h after the LPS administration. Studies carried out on patients treated with interferon alpha (INF-α) showed decreased BDNF circulating levels and increased levels of pro-inflammatory cytokines, such as IL-1 and IL-2, in association with the development of depressive symptoms [78]. Further to this, the acute phases of BD seem to be related to neuroinflammation, with microglial activation and immune cell clusters in the brain [79]. During chronic inflammation, pro-inflammatory cytokines bind microglia, with the release of neurotoxic molecules and a decrease in BDNF signaling [37]. Nevertheless, only a few studies have investigated both BDNF levels and neuroinflammation in samples of BD [80]. The relationship between BDNF, neuroinflammation, and mood episodes should be further investigated in order to improve the knowledge of the pathogenesis of BD.

The majority of studies included in the DNA/mRNA section of our review showed a correlation between BDNF signaling downregulation and BD. In fact, lower circulating BDNF mRNA levels have been found in patients compared to HC. Furthermore, Cinar RK et al. (2016) [45] observed a significant increase in BDNF mRNA levels during the remission period compared with the acute phase. This result can be explained by the fact that mRNA molecules store genetic information that will be decoded and translated into proteins [81]; therefore, decreased mRNA levels reported during acute phases of the disorder can be considered an indirect sign of BDNF levels downregulation. These findings highlight that BDNF may be a biomarker of BD and that its proteolytic conversion may be important in the pathophysiology of BD.

Only one study reported a significant correlation between childhood trauma and BDNF levels [39]. This result, although it requires further investigation, is of particular relevance, since childhood trauma has been reported as one of the most significant risk factors for the development of severe mental disorders and is associated with negative outcomes [82,83], and with a stable dysregulation of other biological pathways, including that of calcium metabolisms—which includes parathormone, vitamin D, and serum levels of calcium—and the hypothalamic–pituitary–adrenal axis [84]. Available research does not allow us to infer the causal relationship between BDNF levels and childhood trauma, but it is possible to hypothesize that low BDNF levels could lead to a reduced resilience to childhood trauma, thus linking trauma to psychopathology.

Another relevant finding is the higher methylation in BDNF gene promoter found in BD patients [48]. Since methylation is a form of epigenetic silencing [85], its presence can be considered an indirect sign of BDNF downregulation.

Only two studies have investigated the Val66Met mutation, also known as the rs6265 SNP. This polymorphism does not seem to alter BDNF biological activity, but it can impair activity-dependent release, resulting in reduced BDNF circulating levels [86]. A correlation between the polymorphism and features that are usually associated with a worse outcome has been found, in line with the hypothesis that the downregulation of BDNF signaling can be associated with a more severe BD course.

Among all articles included in our work, only Zhao et al. (2017) [71] and Skibinska et al. (2021) [55] focused on mature and pro BDNF. While Skibinska et al. found no significant differences, Zhao et al. found decreased mBDNF levels and a lower M/P ratio in BD compared to HC. Interestingly, in a 2013 work, Södersten et al. [87] observed higher mBDNF levels and M/P ratio in mood-stabilized BD patients vs. HC. This heterogeneity in findings could have several explanations, such as differences in mood states or the presence of an effective pharmacological treatment.

It has to be noted that ethnic differences in Val66Met polymorphism [88] and in mature/pro BDNF levels [89] have been reported. However, possible explanations underlying these differences are currently unknown and none of the studies included in this review have addressed this issue. Therefore, further investigations are still needed in order to provide a reasonable explanation of these differences and to assess whether they have an impact on clinical practice.

Taken together, the results of our work show BDNF to be a promising biomarker, with possible future applications in clinical practice. In particular, in the near future, BDNF levels could be used to support the diagnosis of BD, to improve precision in the detection of early stages of BD, and to differentiate between BD and other affective disorders, such as major depressive disorders. In fact, although BD and MDD are distinct clinical entities, they share clinical features, resulting in high rates of misdiagnosis in some cases [90]. As an example, the frequent occurrence of depressive episodes and the later onset of mania in BD subjects may delay a proper diagnosis for years, resulting in greater severity of symptoms, impaired psychosocial functioning, treatment resistance, and higher suicidality [91]. Such a delay is also associated with a higher number of lifetime relapses and hospitalizations, with increased direct and indirect costs associated with the treatment and management of both MDD and BD [92]. Despite increasing knowledge of the pathophysiology of affective disorders, clear clinical indicators and biomarkers for a reliable differential diagnosis between MDD and BD are still missing, which calls for an investigation of novel indices and biomarkers. Future studies are needed in order to identify the predictive role of BDNF in differentiating between MDD and BD. These studies should include the inclusion of patients with affective disorders (MDD and BD) both during acute phases and in euthymic states and should compare their BDNF levels with healthy controls. However, in line with a precision medicine approach, BDNF might be useful in order to predict response to pharmacological treatments in BD patients, since the link between response to treatments and BDNF expression has been reported in some studies included in this review. However, data in this regard are too scarce, and large multicentric studies are needed to support this early evidence.

Our findings should be interpreted in light of some limitations. First, this is not a systematic review; however, we used the most frequently searched scientific databases using generic keywords in order to include as many articles as possible. Furthermore, we did not identify any RCT potentially eligible for inclusion in the review. Additionally, high heterogeneity has been observed regarding sample size, clinical differences among included patients, and data analysis approaches, reducing comparisons among studies. Moreover, we included only papers enrolling patients with BD. It is still unknown if BDNF should be considered as a trans-nosographic marker for more psychiatric disorders, or if it is pathognomonic of a specific condition, such as BD [93,94].

## 5. Conclusions

Taken together, the results of our review show a correlation between the downregulation of BDNF and BD, suggesting a potential role as a biomarker of this neurotrophic factor. In fact, BDNF could be used as a marker for acute BD states and as a marker of clinical response to pharmacological treatments since the normalization of BDNF circulating levels has been found after effective pharmacological treatment. However, this interpretation remains speculative and further studies with larger and less heterogeneous samples are required. Another area open to research is the potential pathogenetic role of BDNF, since its modifications can be a cause or a consequence of BD. Future studies on BDNF and neuroinflammation will clarify the exact mechanism underlying the changes in BDNF serum levels.

## Data Availability

The data presented in this study are available on request from the corresponding author.
